# Bone marrow mesenchymal stem cells derived exosomal miRNAs can modulate diabetic bone-fat imbalance

**DOI:** 10.3389/fendo.2023.1149168

**Published:** 2023-04-14

**Authors:** Fei Han, Chao Wang, Peng Cheng, Ting Liu, Wei-Shan Wang

**Affiliations:** ^1^ Medical College, Shihezi University, Shihezi, Xinjiang, China; ^2^ Department of Orthopaedics, The First Affiliated Hospital of the Medical College, Shihezi University, Shihezi, Xinjiang, China; ^3^ Division of Geriatric Endocrinology, The First Affiliated Hospital, Nanjing Medical University, Nanjing, China; ^4^ Department of Endocrinology, The Affiliated Changsha Central Hospital, Hengyang Medical School, University of South China, Changsha, Hunan, China

**Keywords:** diabetes, osteoporosis, bone-fat imbalance, exosomes, miR-221

## Abstract

**Background:**

Diabetes mellitus is a chronic metabolic disease with systemic complications. Patient with diabetes have increased risks of bone fracture. Previous studies report that diabetes could affect bone metabolism, however, the underlying mechanism is still unclear.

**Methods:**

We isolated exosomes secreted by bone marrow mesenchymal stem cells of normal and diabetic mice and test their effects on osteogenesis and adipogenesis. Then we screened the differential microRNAs by high-throughput sequencing and explored the function of key microRNA *in vitro* and *in vivo*.

**Results:**

We find that lower bone mass and higher marrow fat accumulation, also called bone-fat imbalance, exists in diabetic mouse model. Exosomes secreted by normal bone marrow mesenchymal stem cells (BMSCs-Exos) enhanced osteogenesis and suppressed adipogenesis, while these effects were diminished in diabetic BMSCs-Exos. miR-221, as one of the highly expressed miRNAs within diabetic BMSCs-Exos, showed abilities of suppressing osteogenesis and promoting adipogenesis both *in vitro* and *in vivo*. Elevation of miR-221 level in normal BMSCs-Exos impairs the ability of regulating osteogenesis and adipogenesis. Intriguingly, using the aptamer delivery system, delivery normal BMSCs-Exos specifically to BMSCs increased bone mass, reduced marrow fat accumulation, and promoted bone regeneration in diabetic mice.

**Conclusion:**

We demonstrate that BMSCs derived exosomal miR-221 is a key regulator of diabetic osteoporosis, which may represent a potential therapeutic target for diabetes-related skeletal disorders.

## Background

Diabetes mellitus (DM) is a chronic whole-body metabolic disease leading to a wide range of complications, such as cardiovascular disease, nephropathy, retinopathy, neuropathy, and “sweet bone” disease (1). Epidemiological studies show that the patients with DM have increased risk of bone fracture (2, 3). The hip fracture risk is ranging 2.4- to 7- fold increase in type 1 DM (T1DM) and 2- to 3- times higher in type 2 DM (T2DM) (3, 4). In addition, both bone homeostasis and regeneration are affected in patients with DM (5). Patients with T2DM have lower bone mass, increased bone marrow fat accumulation and impaired ability of bone regeneration, especially in the later stage of T2DM (6). The pathology of diabetic bone degeneration is different from that of postmenopausal and senile osteoporosis which are mainly estrogen-deficiency and aging-related bone loss, respectively. Although previous studies report that diabetes could affect bone metabolism, structure, and strength through effects of obesity, dis-regulated glucose metabolism, toxic effects of glucose oxidized derivatives (advanced glycosylation end products [AGEs]), etc. (7), the underlying mechanism is not fully clear.

Bone marrow mesenchymal stem cells (BMSCs) play a central role in bone homeostasis and regeneration due to their multi-directional differentiation potential and self-renewal ability (8, 9). BMSCs can differentiate into osteoblast and adipocyte according to the genetic and molecular mediators, as well as the Endocrine-disrupting chemicals and local microenvironment (10). During aging, BMSCs are inclined to differentiate into adipocytes rather than osteoblasts, resulting in low bone mass and marrow fat accumulation (11). BMSCs differentiation is also altered in DM condition (12, 13). BMSCs isolated from T1DM animal shows impaired ability of osteogenesis (12). Dysregulated microRNA-491-5p expression in BMSCs isolated from T2DM patients contributes to the reduced ability of osteogenic differentiation (13). High glucose condition regulates MSCs osteogenic differentiation and adipogenic differentiation (14, 15). Interestingly, serum from T2DM patient increases adipogenic differentiation of MSCs (16). In another study, they find that serum miRNAs from T2DM patients, including miR-382-3p and miR-550a-5p, influence osteogenic and adipogenic differentiation of MSCs (17). These studies give us clues that BMSCs lineage fate of osteogenic and adipogenic differentiation could be altered in DM condition and may be regulated by endogenous and exogenous miRNAs coordinately.

Exosomes are between 30 and 150 nm in size, released from multiple cell types, and transported into target cell types through the mechanism of paracrine or endocrine regulation. Exosomes play central roles in intracellular communication by transferring the enriched bioactive molecules, including proteins, mRNAs, and miRNAs, etc. to the target cells. Studies have shown that the exosomes derived from MSCs are involved in a variety of physiological and pathological activities, including bone remodeling and regeneration (18–24). MSC-derived exosomes transfer miR-146a, miR-126, miR-128-3p, and miR-196a to alleviate diabetic osteoporosis and promote fracture healing (20, 23, 25, 26). The contents within exosomes from bone marrow cells altered and the function changed accordingly in pathological conditions. Exosomes secreted by young MSCs have stronger ability of promoting osteogenesis and bone formation than exosomes secreted by old MSCs (27). In DM condition, the contents and function of exosomes also changed. Zhang et al. found that exosomes derived from bone marrow macrophages in T2DM mice impair bone fracture healing through miR-144-5p/Smad1 axis (28). Ning et al. reported that diabetic BMSCs derived exosomes showed lower pro-osteogenic potential than that from normal BMSCs due to the reduced exosomal miR-140-3p level, miR-140-3p-overexpressed-exosomes accelerated bone fracture healing in diabetic rat (29). However, the role of diabetic BMSCs derived exosomes on the linage fate of osteogenesis and adipogenesis remain elusive.

Here we show that exosomes secreted by BMSCs from normal mice enhanced osteogenesis and suppressed adipogenesis, while these effects were diminished in exosomes of BMSCs from diabetic mice. miR-221, as one of the highly expressed miRNAs within in exosomes of diabetic BMSCs, showed abilities of suppressing osteogenesis and promoting adipogenesis. We identified RUNX2 as a direct target of miR-221. Intriguingly, using the aptamer delivery system, specifically delivery normal BMSCs-Exos to BMSCs increased bone formation, reduced marrow fat accumulation, and enhanced bone regeneration in diabetic mice.

## Method and materials

### Mice

Lpr^db/db^ (*db/db*) and Lpr^db/m^ (*db/m*) mice were purchased from Cavens Biogle (Suzhou, China). The animals were housed in a room with a 12-hour light/12-hour dark cycle at 25 degrees Celsius, and free access to sterile food and water. All mice were maintained in the specific pathogen-free facility of the Laboratory Animal Research Center at Shihezi University. Wild type mice used for experiments were allocated randomly. Only male mice were used for experiments. The Animal Care and Use Committee of the Experimental Animal Research Center of Shihezi University reviewed and approved all animal care programs and experiments.

### BMSCs-derived exosome isolation and characterization

We isolated BMSCs-derived exosomes according to the previously described protocol (30). In short, we dissected the femur from diabetic and normal mice, and flushed bone marrow to obtain BMSCs and cultured them at 37°C for 72 hours using α-MEM medium including 10% non-exosomes FBS, 1% penicillin and streptomycin. Next, we collected the culture medium of BMSCs from diabetic and normal mice, respectively. Then, we centrifuged it at 1,000g for 10 min, and filtered its surpernant by a 0.22 mm filter to remove dead cells or debris, the filtered medium was ultracentrifuged at 100,000 g at 4° C for 4-6 h. Finally, the pellet containing exosomes was washed and resuspended in PBS. We characterized exosomes by detecting the expression of its specific markers, such as Calnexin (ab213243, Abcam), CD9 (ab92726, Abcam) and TSG101 (Abcam); we also detected its concentration by NanoSight analysis (Particle Metrix), and observed its size distribution and morphology by transmission electron microscopy (Hitachi H7500 TEM).

### Exosomes uptake assay

BMSCs were cultured in 6-well plates with 2.5×10^6^ cells per well. 4ul PKH26 (sigma) was added to 1ml exosomes suspension, after 5min incubation, 1% BSA was used to stop the reaction system and 1.5ml 10KDa ultracentrifuge tube (Millpore) used to wash off excess PKH26. Then resuspended exosomes in PBS and measured protein concentration using BCA protein assay kit (Beyotime). Add 100ug/ml PKH26-labeled exosomes per well to the well-grown BMSCs and incubated together at 37°C for 12h. Then, fix it with 4% paraformaldehyde for 30 minutes, use PBS to wash the BMSCs for three times, block them with BSA and use phalloidin to mark the cytoskeleton, finally mount the slide with DAPI.

### qRT-PCR analysis

Total RNA was extracted by the TRIzol reagent. Qiagen miRNeasy Mini Kit is used to extract exosomes miRNA. Superscript first-strand synthesis system (Invitrogen) was used to prepare cDNA for qRT-PCR analysis by SYBR Green Master Mix (Qiagen). qRT-PCR analysis of osteogenic and lipogenic genes was performed using the BMSCs which cultured in osteogenic and lipogenic induction medium for 3 days, respectively. The ΔΔCT was used to calculate the mRNA and miRNA expression relative to beta-actin or U6. Primer sequences are listed in **Supplemental Table 1**.

### Western blot

The total cell lysate was separated by SDS-PAGE (sodium dodecyl sulfate polyacrylamide gel electrophoresis) and blotted on a PVDF membrane (polyvinylidene fluoride membrane) (Millipore). Then, the PVDF membrane was blocked with 5% BSA at room temperature for 2 hours, and incubated with specific antibodies against RUNX2 (ab23981, Abcam) ANGPTL2 (37101, SAB) and GAPDH (OT12D9, Origene). The blot was visualized using SuperSignal West Pico PLUS chemiluminescent substrate (SD251210, Thermo Fisher Scientific, Inc).

### Associated-adeno-virus infection

The associated-adeno-virus mmu-miR-221-3p-GFP (AAV-miR-221-3p-GFP) and AAV-miR-NC-GFP with a titer of 1.0E+12 vg/ml were constructed by OBIO co. (Shanghai, China). 30ul AAV-control-GFP and 30ul AAV-miR-221-3p-GFP was injected into bone marrow cavity of each femur of normal mice. Six weeks after virus injection, the mice were anesthetized and sacrificed for specimen collection.

### Conjugation of BMSCs-specific aptamers to BMSCs-exos

We constructed the BMSCs-specific aptamers with an aldehyde group in the 5’ end at Sangon Biotech (Shanghai, China) according to the previously described protocol (31). In short, 200 nM BMSCs-specific aptamers were added to 1.0 mg/mL BMSCs-exos in PBS and rotated at 4°C overnight. Then, Using PBS wash for three times in 10 kDa ultrafiltration tubes to remove unconjugated aptamers. Each mouse was treated with 100ug BMSCs-specific aptamers conjugated BMSCs-exos twice a week *via* tail vein injection.

### *In vivo* image system

Use 1.5ml 10 kDa ultrafiltration tubes to concentrate 1mg/ml BMSCs-Exos and BMSCs-specific aptamers-conjugated-BMSCs-Exos, add 5ul 1mM near-infrared fluorescent dye DIR to the concentrated exosomes and incubated for 30min at 4°C, then using PBS wash for three times in 10 kDa ultrafiltration tubes to remove unconjugated DIR. Mouse was treated with DIR, 100ug DIR-labeled exosomes, 100ug BMSCs-specific aptamers-conjugated-BMSCs-Exos *via* tail vein injection, respectively. Finally, 4h and 12h after injection, we anesthetized the mice for *in vivo* Image system analysis.

### Histochemistry analysis

Femur and tibia were collected after removing surrounding muscle, and fixed in 4% paraformaldehyde for 24 hours, and then decalcified in 10% EDTA for 21 days before being embedded in paraffin. Five-micrometer-thick longitudinally oriented bone sections were stained with H&E to quantify area of adipocytes.

### Immunohistochemistry staining

The bone tissue sections were digested with 0.05% trypsin at 37°C for 15 minutes for antigen retrieval, and then the primary antibody against Osteocalcin (Takara; M173) was incubated overnight at 4°C. Subsequently, the HRP-streptavidin detection system (Dako) was used to detect the immune activity, and the counterstaining was performed with hematoxylin.

### μCT analysis

We use high-resolution μCT for μCT analysis. The voltage of the scanner is set to 65 kV, the current is 153 μA, and the resolution of each pixel is 15 μm. We used image reconstruction software, data analysis software, and 3D model visualization software to analyze the parameters of trabecular bone. Measure the bone volume per tissue volume (BV/TV), trabecular number (Tb.N), trabecular thickness (Tb.Th), trabecular separation (Tb.Sp).

### Osteogenic differentiation and mineralization assay

To induce osteoblastic mineralization, BMSCs were cultured in 6-well plates at 2.5 × 10^6^ cells per well with osteogenic induction medium (α-MEM containing 10% FBS, 10 mM β-glycerolphosphate, 50uM ascorbic acid, and 0.1uM Dexamethasone) for 6-21 days. The culture medium was changed every third day. Then, cells were stained with 2% Alizarin Red S (Sigma-Aldrich) at pH 4.2 to evaluate the cell-matrix mineralization. A Diaphot Inverted Microscope and Camera System (Zeiss) was used for imaging.

### Adipogenic differentiation and lipid drop assay

To induce adipogenic differentiation of BMSCs *in vitro*, BMSCs were cultured in 6-well plates at 2.5 × 10^6^ cells per well with adipogenic induction medium (α-MEM containing 10% FBS, 0.5 mM 3-isobutyl-1-methylxanthine, 5 μg/ml insulin, and 1 μM dexamethasone) for 6-14 days. Culture medium was changed every third day. Then, cells were stained with Oil Red O to detect mature adipocytes. The lipid drop staining was observed by an Inverted Microscope from Zeiss.

### Dual-luciferase reporter gene assay

Wild-type and mutant *Runx2* 3’UTR luciferase reporter gene plasmids were constructed by OBIO co. Then BMSCs were co-transfected WT-*Runx2*-3’UTR, MUT-*Runx2*-3’UTR luciferase reporter gene plasmids together with miR-NC mimic, miR-221 mimic, miR-NC inhibitor and miR-221 inhibitor, respectively. Relative luciferase detection was performed according to technical manual of Dual-luciferase reporter kit (promega) and luminescence intensity was measured at Promega GloMAX Luminescence Detector after 48 h transfection.

### Bone injury model

Bone injury model was established according to previous described protocol. Specifically, mice were anesthetized and the skin were disinfected. Sterility exposes the condyle of each femur, then we make a hole at the intercondylar notch of each femur using a dental drill, finally place a 0.6mm diameters Kirschner wire at the proximal end of the femur. A week after, separate the femur and tibia under aseptic conditions for further micro-CT analysis.

### Statistical analysis

All experiments were repeated three times. Values are expressed as mean ± standard deviation (SD), and the results were analyzed using Prism software. Student’s t-test was used for two-sample experiment, one-way analysis of variance (ANOVA) was used for multiple-sample experiment, non-normally distributed data were tested with non-parametric tests and P<0.05 was considered to be significant.

## Results

### Diabetic mice show imbalanced bone formation and marrow fat accumulation

**Figure 1:**
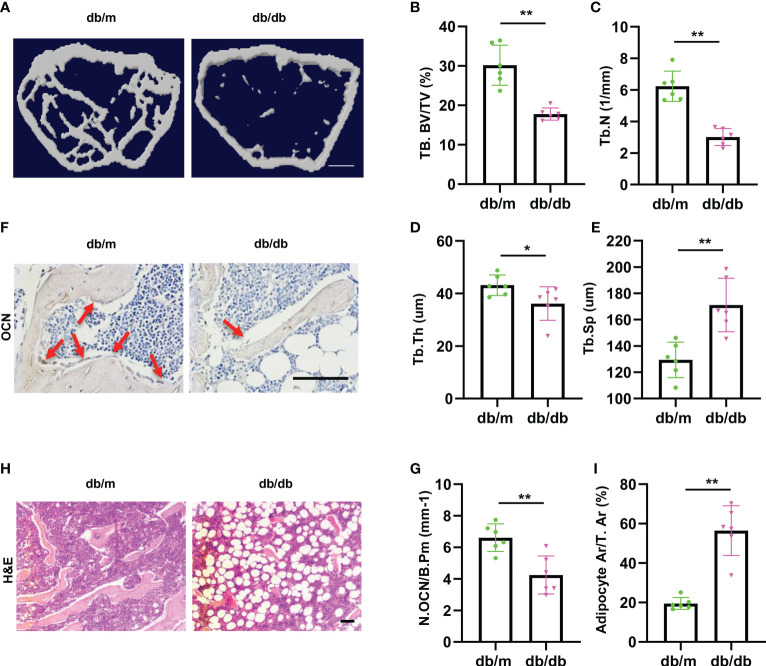
Diabetic mice show imbalanced bone formation and marrow fat accumulation. **(A)** Representative micro-computed tomography (μCT) image of femurs from 3-month male diabetic (db/db) and normal (db/m) mice. **(B-E)** Quantitative μCT analysis of trabecular bone volume **(B)**, Trabecular bone number **(C)**, Trabecular thickness **(D)** and Trabecular separation **(E)**. Scale bar: 1 mm **(F, G)** Representative images of osteocalcin immunohistochemical staining **(F)** and number of osteoblasts in the distal femur **(G)**, red arrows represent osteocalcin-positive–staining cells. Scale bar: 100 μm. **(H, I)** Representative images of H&E staining **(H)** and area of adipose cells in the distal femur **(I)**. Scale bar: 100 μm. The data is shown as the mean ± SD. * P <0.05, ** P <0.01, Welch’s t test is used in **(B)** and **(I)**, Student’s t-test used for other figures, n=6 per group.

To investigate the pathology of diabetes-related osteoporosis, we take the advantage of leptin-receptor-deficient mice (*db/db*), a mouse model for T2DM, to analyze bone phenotype. First, we analyzed the bone phenotype of *db/db* mice (hereafter diabetic mice) and *db/m* mice (hereafter normal mice). Micro-CT analysis showed that diabetic mice had lower trabecular bone volume and number, but higher trabecular separation compared with control mice (**Figures 1A–E**). The immunohistochemically analysis revealed the number of osteocalcin-positive osteoblasts on the trabecular bone surface was decreased in diabetic mice compared to that from normal mice (**Figures 1F, G**). Hematoxylin-eosin staining showed that the area of adipocytes in the bone marrow were increased in diabetic mice compared to that of normal mice (**Figures 1H, I**), indicating the bone-fat imbalance exits in diabetes-related osteoporosis.

These data indicate that the diabetic mice showed lower osteoblastic bone formation and increased marrow fat accumulation.

### BMSCs derived exosomes from diabetic mice showed impaired ability of promoting osteogenesis and inhibiting adipogenesis

**Figure 2:**
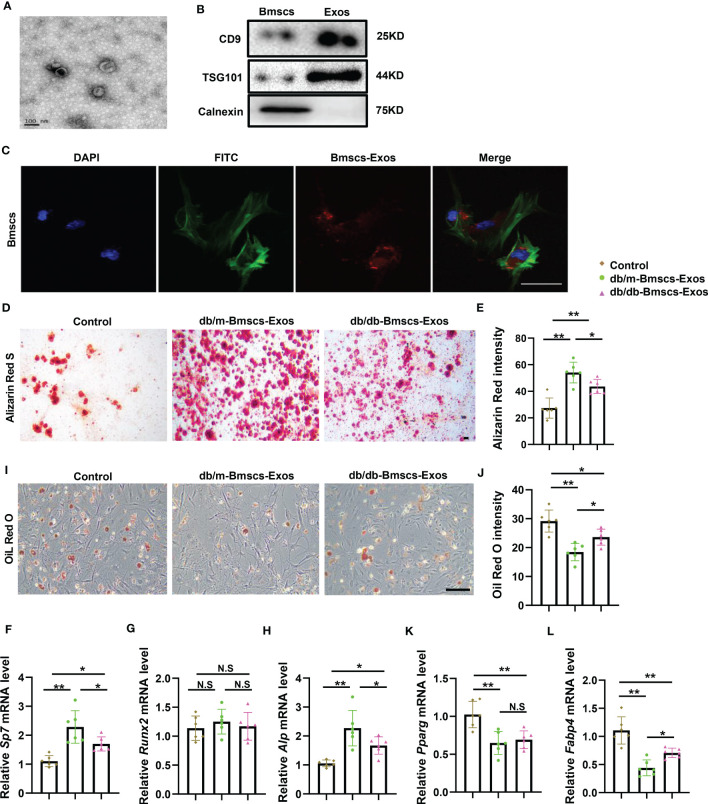
BMSCs derived exosomes from diabetic mice showed impaired ability of promoting osteogenesis and inhibiting adipogenesis. **(A)** Electron microscopy (sem) images of BMSCs-derived Exosomes (BMSCs-Exos) collected by ultracentrifugation. Scale bar: 100 nm. **(B)** Western blot of Exosomes markers, CD9, TSG101 and calnexin, full-length blots are presented in **Supplementary Figure 1A-C**. **(C)** 100ug/ml BMSCs-Exos was added to BMSCs and incubated for 12h. The representative image of BMSCs uptake the BMSCs-Exos. Blue fluorescence marks the DAPI-labeled nucleus, green fluorescence marks phalloidin-labeled cytoskeleton, red fluorescence marks PKH26-labeled BMSCs-Exos. Scale bar: 100um. **(D-L)** Staining of BMSCs-Exos from diabetic mice and normal mice cultured in osteogenic induction medium and adipogenic induction medium for 14 days. qRT-PCR analysis of BMSCs-Exos from diabetic mice and normal mice cultured in osteogenic induction medium and adipogenic induction medium for 3 days. The representative images of matrix mineralized ash **(D)** and quantification of Alizarin Red S staining **(E)**, Scale bar: 100 μM; mRNA expression levels of osteogenic gene, *Sp7*, *Runx2* and *Alp*, respectively **(F-H)**, n=6 per group. Representative images of lipid droplet, Scale bar: 100 μM **(I)** and quantification of Oil Red O staining **(J)**; mRNA expression levels of adipogenic gene, *Pparg* and *Fabp4*, respectively **(K, L)**, n=6 per group. The data is shown as the mean ± SD. * P <0.05, ** P <0.01, NS., not significant (ANOVA).

The above bone phenotype of imbalanced bone formation and marrow fat accumulation indicate that osteogenesis and adipogenesis may be affected in diabetes. As exosomes play central roles in intracellular communication through the mechanism of paracrine or endocrine regulation. Thus, we investigated the role of diabetic BMSCs derived exosomes on osteogenesis and adipogenesis. BMSCs derived exosomes (BMSCs-Exos) from diabetic mice and normal mice were collected and tested their effects on osteoblastic and adipogenic differentiation of BMSCs. Firstly, the BMSCs-Exos were obtained by ultracentrifugation after removing the dead cells and debris from the conditional medium. By using electron microscopy, we observed the exosomes were round-shaped vesicles with bilayer membrane structure. Western blotting analysis showed high level of exosome-specific protein markers (CD9 and TSG101) in the BMSCs-Exos and negative expression endoplasmic reticulum preotein Calnexin (**Figures 2A, B**). Next, we tested whether BMSCs-Exos can be transferred into BMSCs. Red fluorescent PKH26 was mixed with BMSCs-Exos to trace the transportation. The appearance of the red fluorescent dye in the receipt cells indicates that BMSCs-Exos can be taken up by BMSCs (**Figure 2C**). Next, we detected the effects of normal BMSCs-Exos and diabetic BMSCs-Exos on osteogenesis and adipogenesis. The expression of osteogenesis-related genes, including *Alp, Sp7*, and *Runx2*, as well as the mineralization of BMSCs, were all increased in the normal BMSCs-Exos treated group compared to PBS treated control group as determined by QPCR analysis and Alizarin Red staining (**Figures 2D–H**). Furthermore, the normal BMSCs-Exos treated group showed reduced expression of adipogenesis-related genes, such as *Pparg* and *Fabp4*, and fewer lipid droplet formation as measured by Oil Red staining compared to PBS treated control (**Figures 2I–L**). While these effects on osteogenesis and adipogenesis were all diminished in diabetic BMSCs-Exos.

These data suggest that normal BMSCs-Exos can be taken up by BMSCs and thus enhance osteogenesis and suppress adipogenesis, while these effects were diminished in diabetic BMSCs-Exos.

### Diabetic BMSCs derived exosomal miR-221 suppresses osteogenesis and enhances adipogenesis

**Figure 3:**
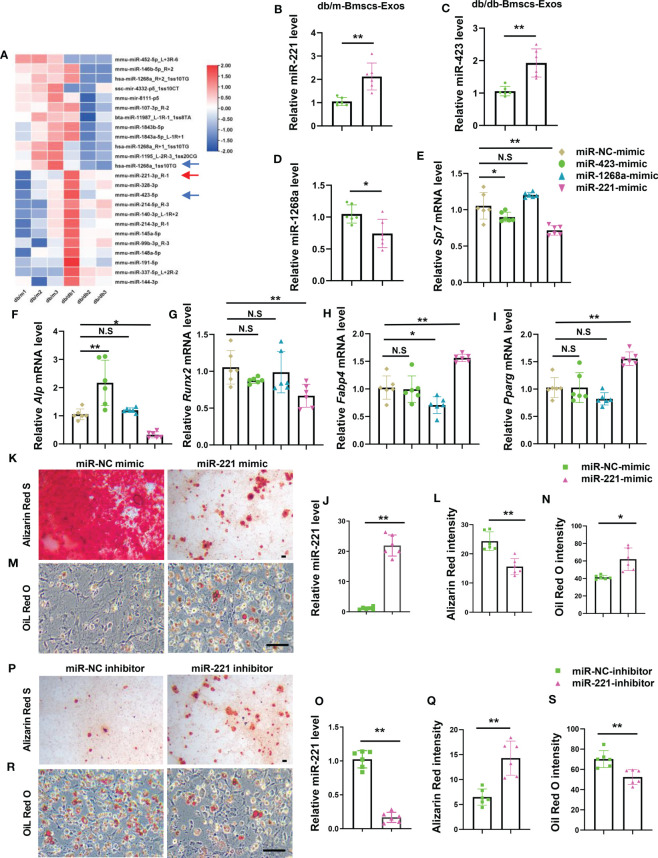
Diabetic BMSCs derived exosomal miR-221 suppresses osteogenesis and enhances adipogenesis. **(A)** Microarray analysis of deregulated miRNAs in normal and diabetic mice BMSCs Exosomes, n=3 per group. **(B-D)** qRT-PCR analysis of the expression of miR-221 **(B)**, miR-423 **(C)**, and miR-1268a **(D)** in BMSCs Exosomes derived from normal and diabetic mice, n=6 per group. **(E-I)** Over-express miR-221, miR-423, and miR-1268a in BMSCs from wild type mice in C57 BL/6J background for 6 hours, respectively, then culture them in osteogenic induction medium and adipogenic induction medium for 3 days. Relative mRNA expression level of osteogenesis-related genes *Sp7*, *Alp* and *Runx2*, respectively **(E-G)**; mRNA expression levels of adipogenic gene, *Fabp4* and *Pparg*, respectively **(H, I)**; NC, normal control, n=6 per group. **(J-N)** Transfected miR-221 mimic into BMSCs for 6 hours, then culture them in osteogenic induction medium for 21 days and adipogenic induction medium for 8 days. miRNA expression levels of miR-221 **(J)**; representative images of matrix mineralized ash **(K)**, Scale bar: 100 μM; quantification of Alizarin Red S staining **(L)**; Representative images of lipid droplet **(M)**, Scale bar: 100 μM; quantification of Oil Red O staining **(N)**. NC, normal control, n=6 per group. **(O-S)** Transfected miR-221 inhibitor into BMSCs for 6 hours, then culture them in osteogenic induction medium and adipogenic induction medium for 14 days. miRNA expression levels of miR-221 **(O)**; representative images of matrix mineralized ash **(P)**, Scale bar: 100 μM; quantification of Alizarin Red S staining **(Q)**; Representative images of lipid droplet **(R)**, Scale bar: 100 μM; quantification of Oil Red O staining **(S)**. NC, normal control, n=6 per group. The data is shown as the mean ± SD. * P <0.05, ** P <0.01, Welch’s t test is used in **(B, C)** and **(M)**, Student’s t-test is used in **(D, K, O)** and **(Q)**, Kruskal wallis test used in **(G)**, ANOVA used in others. NS, not significant.

To determine the key miRNA/miRNAs within BMSCs-Exos that regulate BMSCs lineage fate switch. BMSCs-Exos of diabetic mice and normal mice were isolated to identify dysregulated miRNAs by performing miRNA array (**Figure 3A**). Among them, miR-221-3p, miR-423-5p, and miR-1268a were abundant within the exosomes and showed a large difference in expression between diabetic and normal BMSCs-Exos, and thus were chosen for further study. The difference of their levels between diabetic and normal BMSCs-Exos was further confirmed by qPCR. The level of miR-221-3p and miR-423-5p was low in normal BMSCs-Exos but was high in diabetic BMSCs-Exos, while miR-1268a level was lower in diabetic BMSCs-Exos than normal BMSCs-Exos (**Figures 3B–D**). Next, we evaluate the effects of these three candidates on osteogenesis and adipogenesis. We found that, among these three candidates, miR-221-3p (hereafter miR-221) showed the ability of suppressing osteogenesis and enhancing adipogenesis as evidenced by lower expression of osteogenesis-related genes, including *Alp*, *Runx2*, and *Sp7*, but higher expression of adipogenesis-related genes, such as *Pparg* and *Fabp4* in miR-221 mimic treated BMSCs compared to control mimics treated group (**Figures 3E–I**).

To further test the effects of miR-221 on osteogenesis and adipogenesis, BMSCs were transfected with miR-221 mimics or miR-221 inhibitor and underwent osteoblastic and adipogenic differentiations, respectively. The levels of miR-221 in BMSCs were significantly increased or decreased after BMSCs were transfected with miR-221 mimic or miR-221 inhibitor (**Figures 3J, O**). Overexpression of miR-221 inhibited, while silencing of miR-221 promoted, osteogenic differentiation of BMSCs as measured by Alizarin Red staining (**Figures 3K, L, P, Q**). In contrast, overexpression of miR-221 promoted, while silencing of miR-221 suppressed, lipid droplet formation in BMSCs determined by Oil Red O staining (**Figures 3M, N, R, S**).

These data showed that miR-221 inhibited osteogenic differentiation and promoted adipogenic differentiation in BMSCs, of which the level was high in diabetic BMSCs-Exos.

### Overexpression of miR-221 in normal mice showed bone loss and marrow fat accumulation

**Figure 4:**
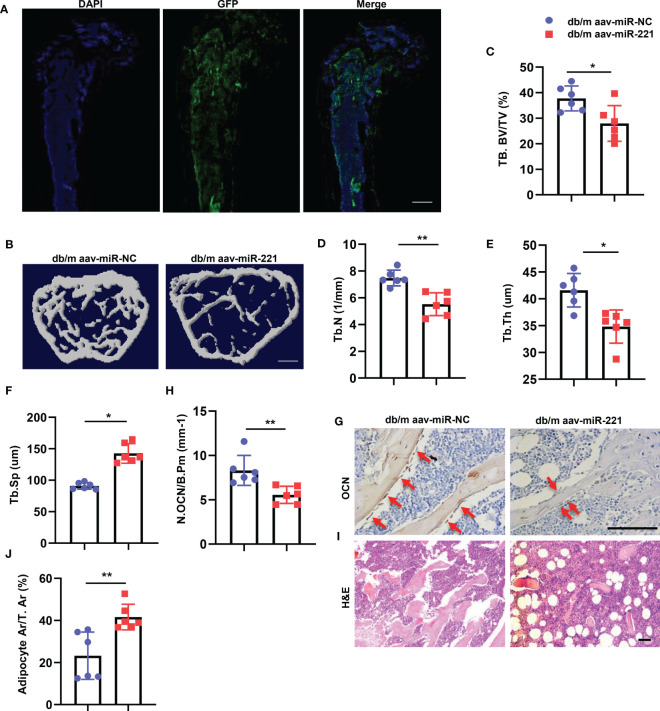
Overexpression of miR-221 in normal mice showed bone loss and marrow fat accumulation. **(A-F)** Intramedullary injection of 30ul 1.0E+12 vg/ml AAV-microRNA-NC-EGFP and AAV-microRNA-221-EGFP in normal mice, six weeks after AAV injection, the femur were collected for further analysis. Representative fluorescent images of GFP expression, blue fluorescence marks the DAPI-labeled nucleus and green fluorescence indicates successful virus injection **(A)**. Scale bar: 5mm; Representative micro-computed tomography (μCT) image **(B)**; Quantitative μCT analysis of trabecular bone volume **(C)**, Trabecular bone number **(D)**, Trabecular thickness **(E)** and Trabecular separation **(F)**, NC, normal control, n=6 per group. Scale bar: 1mm. **(G, H)** Representative images of osteocalcin immunohistochemical staining **(G)** and number of osteoblasts in the distal femur **(H)**, red arrows represent osteocalcin-positive–staining cells. Scale bar: 100 μm. **(I, J)** Representative images of H&E staining and area of adipose cells in the distal femur, respectively. Scale bar: 100 μm. The data is shown as the mean ± SD. * P <0.05, ** P <0.01 (ANOVA).

The above data indicate that high level of miR-221 within the exosomes may contribute to the impaired bone anabolic effects of diabetic BMSCs-Exo leading to diabetic osteoporosis. To investigate whether elevation of miR-221 level could lead to bone loss and marrow fat accumulation in normal mice, normal mice were received intramedullary injection of adeno-associated viral-miR-221-GFP (AAV-miR-221, 10^^12^vg/ml, 30ul) for 6 weeks, AAV-miR-NCs-GFP was served as control. Green fluorescence signal was detected in bone marrow cells which indicated the successful transfection of AAV-miR-221 (**Figure 4A**). Next, we analyzed the bone phenotype of the normal mice with overexpression of miR-221. Trabecular bone volume and number were decreased, and trabecular separation was increased in normal mice treated with AAV-miR-221 compared with AAV-miR-NC-treated control (**Figures 4B–F**). Mice with AAV-miR-221 treatment showed lower number of osteoblasts on trabecular bone surface, but higher area of adipocytes compared to AAV-miR-NC treated control (**Figures 4G–J**).

Taken together, these results indicate that overexpression of miR-221 leads to bone loss and marrow fat accumulation in normal mice.

### MiR-221 regulates osteogenesis and adipogenesis by direct targeting RUNX2

**Figure 5:**
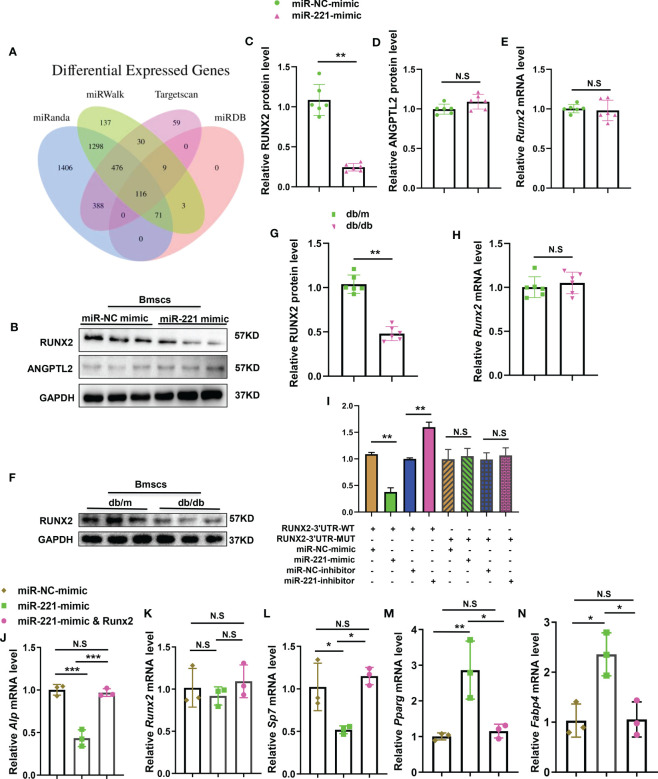
MiR-221 regulates osteogenesis and adipogenesis by direct targeting Runx2. **(A)** Bioinformatics tools (miRanda, miRwalk, targetscan and miRDB) were used to predict the targets of miR-221. **(B)** Representative Western blot image of 3 independent experiments in BMSCs transfected with miR-NC mimic and miR-221mimic, GAPDH was used as a loading control, full-length blots are presented in **Supplementary Figure 2A-C**, n=3 per group; **(C, D)** Quantification of the relative protein levels of RUNX2 **(C)** and ANGPTL2 **(D)**, n=6 per group; **(E)** Quantification of the relative mRNA levels of *Runx2* in BMSCs transfected with miR-NC mimic and miR-221mimic, n=6 per group. **(F-H)** Extract primary BMSCs RNA and protein from normal and diabetic mice for quantitative analysis of RUNX2 expression; **(F)** Representative Western blot image of 3 independent experiments in normal and diabetic BMSCs, full-length blots are presented in **Supplementary Figure 3A, B**, n=3 per group; **(G)** Quantification of the relative protein levels of RUNX2, n=6 per group; **(H)** Quantification of the relative mRNA levels of *Runx2* in normal and diabetic BMSCs, n=6 per group. **(I)** BMSCs were transfected with luciferase reporter carrying WT or MUT 3′-UTR of the *Runx2* gene, effects of miR-221mimic and miR-221inhibitor on the reporter constructs were determined at 48 hours after transfection. Quantification of relative luciferase activity of WT–*Runx2*–3′-UTR, MUT–*Runx2*-3′-UTR respectively, n = 3 per group. **(J-N)** Transfected miR-221 and miR-221 together with Runx2 into BMSCs for 6 hours, then culture them in osteogenic induction medium and adipogenic induction medium for 3 days. Relative mRNA expression level of osteogenesis-related genes *Alp*, *Runx2* and *Sp7*, respectively **(J-L)**; mRNA expression levels of adipogenic gene, *Pparg* and *Fabp4*, respectively **(M, N)**; NC, normal control, n=3 per group. The data is shown as the mean ± SD. * P <0.05, ** P <0.01, Student’s t-test is used in Figures C–H and ANOVA is used in Figures **(I)** NS, not significant.

To test the direct targets of miR-221, we used bioinformatics tools TargetScan, miRDB, miRWalk, and miRanda (32) to predict the targets of miR-221. Among the predicted genes (**Figure 5A**), we chose *Runx2*, which had been reported to participate in regulating osteogenesis and adipogenesis (33–36), and *Angptl2* for further verification. Overexpression of miR-221 decreased the levels of RUNX2 proteins, but not ANGPTL2, in BMSCs compared to miR-NC treated group (**Figures 5B–D**). While the mRNA expression of *Runx2* was not significantly change between these two groups (**Figure 5E**). Moreover, the level of RUNX2 in BMSCs from normal mice and diabetic mice was detected respectively. The protein level of RUNX2 in BMSCs from diabetic mice was decreased, while the mRNA level of *Runx2* was not significantly changed, compared to that of normal mice (**Figures 5F–H**). These data indicate that miR-221 may post-transcriptionally regulate *Runx2* expression.

To investigate whether miR-221 directly targets RUNX2, luciferase reporter constructs containing the predicted miRNA-binding site of *Runx2* (WT-PGL3-*Runx2*) or mutated nucleotides within the binding site (MUT-PGL3-*Runx2*) were generated. We transfected WT-PGL3-*Runx2* or MUT-PGL3-*Runx2* with co-transfection of miR-221 mimic or miR-221 inhibitor and measured the effects of miR-221 on luciferase translation by detecting luciferase enzyme activity. Overexpression of miR-221 suppressed the luciferase activity of the WT-PGL3-*Runx2* 3’-UTR reporter genes. While the mutation of nucleotides within the sequences of the putative target site in the 3’-UTR of *Runx2* eliminated this repression, confirming the direct targeting of miR-221 on 3’-UTR of *Runx2* genes (**Figure 5I**). Meanwhile, overexpression of *Runx2* in miR-221 transfected BMSCs neutralized the inhibition effect of miR-221 on osteogenesis and promotion effect on adipogenesis (**Figures 5J–N**).

The above data imply that *Runx2*, as the direct target of miR-221, mediates the effects of miR-221 on regulating osteogenesis and adipogenesis.

### MiR-221 lowered diabetic-BMSCs-Exos regains the ability of enhancing osteogenesis and suppressing adipogenesis

**Figure 6:**
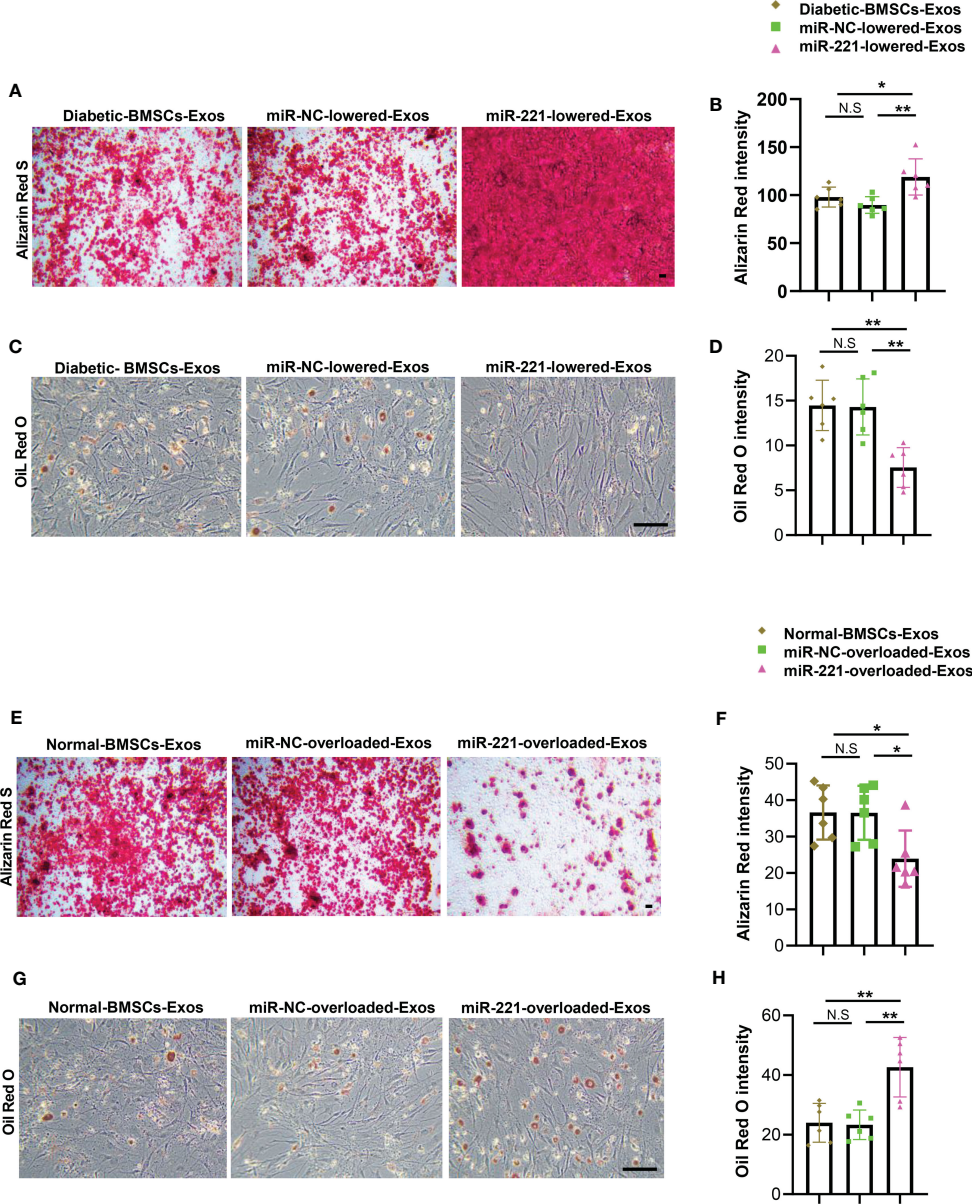
MiR-221 lowered diabetic-BMSCs-Exos regains the ability of enhancing osteogenesis and suppressing adipogenesis. **(A-D)** Transfected miR-221 inhibitor in BMSCs of diabetic mice using lipofectamine™ 2000 ragent for 72h, isolated miR-221-lowered-exosomes, then add to BMSCs which cultured in osteogenic and adipogenic medium. **(A-D)** miR-221-lowered-exosomes were added to BMSCs, then cultured BMSCs in osteogenic induction medium for 21 days, and BMSCs in osteogenic induction medium for 14 days. **(A)** Representative images of matrix mineralized ash, Scale bar: 100 μM; **(B)** quantification of Alizarin Red S staining; **(C, D)** Representative images of lipid droplet, Scale bar: 100 μM and quantification of Oil Red O staining. **(E-H)** Overexpress miR-221 in normal BMSC, collect miR-221-overloaded-Exos and add to BMSCs which cultured in osteogenic and adipogenic medium for 21 days and 14 days, respectively. **(E)** Representative images of matrix mineralized ash, Scale bar: 100 μM; **(F)** quantification of Alizarin Red S staining; **(G, H)** Representative images of lipid droplet, Scale bar: 100 μM and quantification of Oil Red O staining. The data is shown as the mean ± SD. * P <0.05, ** P <0.01 (ANOVA). NS, not significant.

The above data showed that miR-221 may be the key effector within the BMSCs-Exo that regulate osteogenesis and adipogenesis. Next, we investigated whether alteration of miR-221 level within BMSCs-Exo could change the effects on osteogenesis and adipogenesis. First, we transfected diabetic BMSCs with miR-221 inhibitor and collected the miR-221-lowered-exosomes. BMSCs were induced into osteoblastic and adipogenic differentiation respectively with addition of miR-221-lowered-diabetic BMSCs-Exos or diabetic BMSCs-Exos treatment. BMSCs treated with miR-221-lowered-diabetic BMSCs-Exos showed more mineralization, but fewer lipid droplet formation compared to diabetic BMSCs-Exos treated BMSCs (**Figures 6A–D**).

To further investigate the effects of endogenous miR-221 within normal BMSCs-Exos on osteogenesis and adipogenesis. We transfected normal BMSCs with miR-221 mimics to collect miR-221-overloaded-normal BMSCs-Exos. BMSCs were induced into differentiation of osteoblasts and adipocytes respectively with addition of miR-221-overloaded-normal BMSCs-Exos or normal BMSCs-Exos treatment. BMSCs treated with miR-221-overloaded-normal BMSCs-Exos showed decreased osteogenesis as determined by impaired mineralization (**Figures 6E, F**), but enhanced adipogenesis as determined by more lipid droplet formation compared to normal BMSCs-Exos treated BMSCs (**Figures 6G, H**).

All these results suggest that overexpression of miR-221 within normal BMSCs-Exos impairs, while lowering miR-221 level within diabetic BMSCs-Exos regains, the ability on regulating osteogenesis and adipogenesis.

### Aptamer-BMSCs-Exos alleviated bone-fat imbalance in diabetic mice

**Figure 7:**
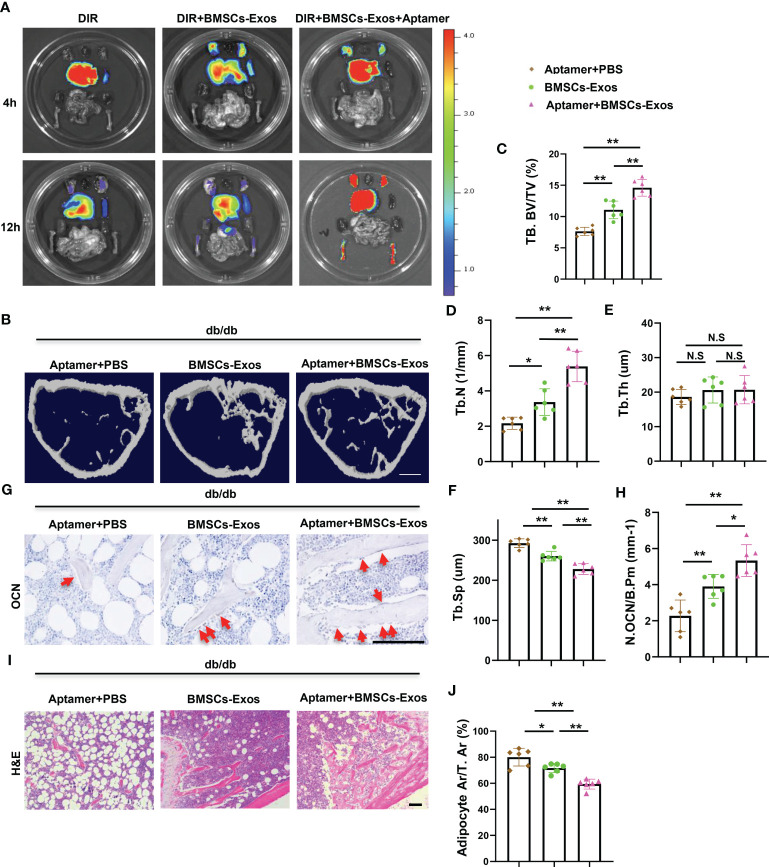
Aptamer-BMSCs-Exos alleviated bone-fat imbalance in diabetic mice. **(A, B)** Conjugate normal BMSCs-Exos with BMSCs-specific aptamer and use near-infrared fluorescent dye DIR to label BMSCs-Exos, then inject 100ug modified exosomes to diabetic mice *via* tail vein. **(A)** Representative FMT images of the near-infrared fluorescence signals in organs isolated from mice administered with DIR dye alone, DIR-labeled BMSCs-Exos (DIR-BMSCs-Exos) or aptamer conjugated DIR-labeled BMSCs-Exos (DIR-BMSCs-Exos-Apt) for 4h (up panel) and 12h (bottom panel). **(B)** Inject 100ug modified exosomes to diabetic mice *via* tail vein twice a week, representative μCT images of diabetic mouse femora. Scale bar: 1mm. **(C-F)** Quantitative μCT analysis of trabecular bone volume **(C)**, Trabecular bone number **(D)**, Trabecular thickness **(E)** and Trabecular separation **(F)**. **(G, H)** Representative images of osteocalcin immunohistochemical staining **(G)** and number of osteoblasts in the distal femur **(H)**, red arrows represent osteocalcin-positive–staining cells. Scale bar: 100 μM. **(I, J)** Representative images of H&E staining **(I)** and area of adipose cells in the distal femur **(J)**. Scale bar: 100 μM. The data is shown as the mean ± SD, n = 6 per group, * P < 0.05 ** P < 0.01 (ANOVA), n=6 per group. NS, not significant.

To further investigate the therapeutic potential of BMSCs-Exos on diabetes-related osteoporosis *in vivo*, normal BMSCs-Exos was conjugated with BMSC-targeting aptamer and injected into diabetic mice *via* tail vein. We first tested the efficiency of this system. BMSCs-Exos were conjugated with the aptamer (BMSCs-Exos-Apt), and then labeled with near-infrared fluorescent dye DIR for 30 min at 4°C, and the excess unconjugated aptamer and DIR were removed by centrifugation in a 10 kDa ultracentrifuge tube to obtain a DIR-BMSCs-Exos-aptamer complex (DIR-BMSCs-Exos-Apt). After intravenous injection of an equal amount (100ug) of DIR-BMSCs-Exos-Apt and DIR-labeled BMSCs-Exos (DIR-BMSCs-Exos) for 4 or 12 h, a fluorescence molecular tomography imaging system was used to examine the tissue distribution of DIR-BMSCs-Exos-Apt. The majority of DIR-BMSCs-Exos accumulated in the liver, but rare and weak signals were detected in the limbs at 4 and 12 hours after injection respectively. Of note, stronger fluorescence signals were observed in the limbs of the DIR-BMSCs-Exos-Apt treated mice at 12 hours after injection, indicating that more BMSCs-Exos were transported into bone tissue with conjugation of BMSCs-aptamer (**Figure 7A**). Next, we tested the effects of BMSCs-aptamer conjugated normal BMSCs-Exos (Apt-BMSCs-Exos) on diabetic osteoporosis. Diabetic Mice treated with Apt-BMSCs-Exos showed significant higher trabecular bone volume, number, but lower trabecular separation, compared with BMSCs-Exos treated mice (**Figures 7B–F**). In addition, we observed a higher number of osteoblasts on trabecular bone surfaces and a lower area of adipocytes in the bone marrow in Apt-BMSCs-Exos treated mice compared with BMSCs-Exos treated mice. (**Figures 7G–J**).

These results indicate that delivery normal BMSC-Exos to BMSCs using aptamer system increased bone formation and reduced bone marrow fat accumulation in diabetic mice.

### Aptamer-BMSCs-Exos promoted bone regeneration in diabetic mice

**Figure 8:**
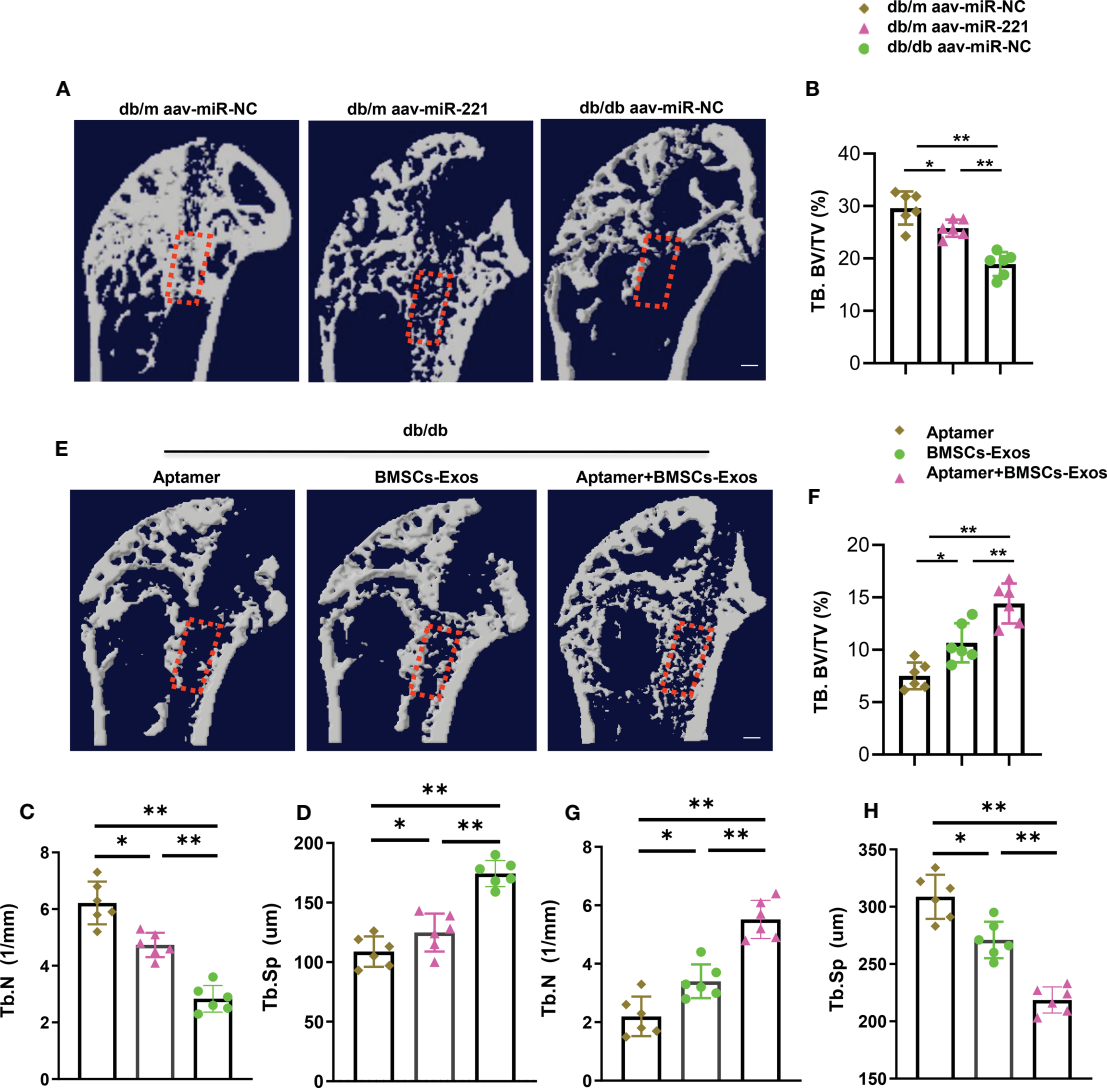
Aptamer-BMSCs-Exos promoted bone regeneration in diabetic mice. Using a dental drill to place a 0.6mm diameters Kirschner wire at the proximal end of the femur. A week after, separate the femur and tibia under aseptic conditions for further micro-CT analysis. **(A, E)** Representative micro-CT images; Scale bar: 1mm. **(B-D, F-H)** Quantitative analysis of bone regeneration after femoral trabecular bone ablation of 3-month-old diabetic and normal mice. Selected areas for the measurements of bone volume/tissue volume (BV/TV) were indicated with a red square. The data is shown as the mean ± SD. n = 6 per group, * P <0.05 ** P < 0.01, (ANOVA).

To investigate the effects of Aptamer-BMSCs-Exos on bone damage repair, we next generated bone regeneration mouse model by surgical ablation of the trabecular bone in diabetic mice. Micro-CT analysis showed the bone volume and trabecular bone number in the bone regeneration area was lower and the trabecular bone separation was higher in diabetic mice than that in normal mice indicating that bone regeneration ability was impaired in diabetic condition, which is consistent with other’s report (**Figures 8A–D**) (1). First, we tested whether elevation of miR-221 could impair bone regeneration in normal mice. Intramedullary injection of AAV-miR-221 decreased the bone volume in the bone regeneration area in normal mice compared with AAV-miR-NC treated control (**Figures 8A–D**). Next, we measured the effects of the Aptamer-BMSCs-Exos on bone regeneration. Tail vein injection of Aptamer-BMSCs-Exos dramatically increased bone volume, trabecular bone number and decreased bone separation in the regenerated area in diabetic mice compared to BMSCs-Exos control group (**Figures 8E–H**).

These results indicate that normal BMSCs-Exos could promote bone regeneration in diabetic mice.

## Discussion

In this study, we found that exosomes secreted by normal BMSCs showed strong ability of enhancing osteogenesis and repressing adipogenesis, while these effects were all diminished in diabetic BMSCs derived exosomes. Through miRNAs high profile screening, we found high level of miR-221 within diabetic BMSCs-Exos may contribute to impaired ability on osteogenesis and adipogenesis. Lowering miR-221 level in diabetic BMSCs-Exos recover the ability of enhancing osteogenesis and suppressing adipogenesis, leading to alleviated bone loss and marrow fat accumulation in diabetic mice. We identified RUNX2 as a direct target of miR-221. Intriguingly, using the aptamer delivery system, specifically delivery normal BMSCs-exosomes to BMSCs increased bone formation, reduced marrow fat accumulation, and enhanced bone regeneration in diabetic mice. Thus, our findings indicate that BMSCs derived exosomal miR-221 is a key regulator of osteogenesis and adipogenesis, which may represent a potential therapeutic target for diabetic-related bone loss.

In this study, we reported that the bone-fat imbalance exists in skeleton of diabetic mice, as characterized by decreased bone mass and increased marrow fat accumulation, consistent with previous report (37–40). Previous studies reported that endogenous miR-188 and lncRNA-Bmncr regulate BMSCs lineage fate between osteoblasts and adipocytes during aging (9, 41). In this study, we measured the altered miRNAs within the diabetic and normal BMSCs-Exos, while miR-188 was undetectable in neither diabetic BMSCs-Exos nor normal BMSCs-Exos. The explanations for these data are, first, the factors that responsible for BMSCs lineage fate switch between aged mice and diabetic mice may be different due to the distinguished pathological microenvironments; second, miR-188 may regulate BMSCs differentiation mainly in endogenous manner, but not in exosomes manner.

MicroRNAs within the exosomes have attracted more attention due to the high abundance within exosomes and powerful functions in cellular communications (42). BMSCs derived exosomes play vital roles in bone homeostasis. MSC-derived exosomes transfer miR-126 to promote fracture healing (25). Exosomes secreted by young MSCs promote osteogenesis and bone formation in older rats (27). Zhang et al. has found that exosomes derived from BMSCs in T1DM mice have a lower osteogenic ability than that of normal mice (28). Here we firstly report BMSCs-Exos from in diabetic mice showed impaired ability of enhancing osteogenesis and suppressing adipogenesis. In addition, we screen the altered miRNAs within the BMSCs-Exos, miR-221 shows the ability of suppressing osteogenesis while enhancing adipogenesis. Thus, we choose miR-221 for the following study. However, we can’t exclude the possibilities that other miRNAs, lncRNAs, DNAs, or proteins are also involved in the altered function of diabetic BMSCs-Exos on osteogenesis and adipogenesis. In the present study, the finding that miR-221-overloaded exosomes showed impaired ability of enhancing osteogenesis and inhibiting adipogenesis suggests that elevated miR-221 level within exosomes play vital roles in diabetic osteoporosis. However, we did not test what factor/factors could regulate miR-221 level within diabetic BMSCs-Exos. Previous studies report that dis-regulated glucose metabolism, toxic effects of glucose oxidized derivatives could affect bone metabolism, it is an interesting topic to investigate whether diabetic BMSCs could sense or response to the glucose oxidized derivatives, such as AGEs, and result in the alteration of contents and function of exosomes.

Previously, it has been reported that miR-221 regulates osteogenic differentiation and autophagy (43–45). However, the role of exosomal miR-221 in determining the BMSCs lineage fate of osteogenic and adipogenic differentiation, is firstly reported in this study. Consistent with our result, Gan et al. reported that, in high glucose condition, silencing miR-221 and miR-222 promotes osteogenic differentiation of BMSCs through IGF-1/ERK pathway (46). In another study, Maeda et al, found that Synovium-Derived miRNA-221 is increased in rheumatoid arthritis and transfecting miR-221-3p into mouse primary calvaria osteoblasts inhibits osteoblast differentiation and mineralization (47). In this study, our data show that miR-221 inhibits osteogenic differentiation of BMSCs and enhances adipogenesis. However, the mouse models of transgenic overexpressing miR-221 or knockout of miR-221 in skeletal progenitors could be a better way to test the role of miR-221 on osteogenic differentiation *in vivo*.

We demonstrate that miR-221 directly targets *RUNX2* gene. Consist with the previous studies that show the RUNX2 is involved in adipogenesis and adipogenesis (41, 48–50). We realize that direct targets of miR-221 on regulating osteogenesis and adipogenesis, other than RUNX2, may exist and need to be characterized.

Aptamers are single-stranded nucleic acid molecules that bind to the target by folding into a three-dimensional structure with high affinity and selectivity (31, 51, 52). Our results indicate that treatment of normal BMSCs-Exos with BMSCs-specific aptamers increase bone formation in diabetic mice and reduce bone marrow fat accumulation and may be a new therapeutic target for the treatment of diabetic osteoporosis.

## Conclusion

We demonstrate that BMSCs derived exosomal miR-221 is a key regulator of diabetic osteoporosis, which may represent a potential therapeutic target for diabetes-related skeletal disorders.

## Data availability statement

The original contributions presented in the study are included in the article/**Supplementary Material**, further inquiries can be directed to the corresponding author/s.

## Ethics statement

The animal study was reviewed and approved by The Animal Care and Use Committee of the Experimental Animal Research Center of Shihezi University.

## Author contributions

W-SW, TL, PC, and FH designed the experiments; FH, carried out most of the experiments; CW helped to conduct animal experiments and collect the samples; W-SW, PC, and TL supervised the experiments, analyzed results, and wrote the manuscript. All authors contributed to the article and approved the submitted version.
