# Supplementary material for: Bone marrow mesenchymal stem cells derived exosomal miRNAs can modulate diabetic bone-fat imbalance
Source: Front Endocrinol (Lausanne). 2023 Apr 14;14:1149168. doi: 10.3389/fendo.2023.1149168 (PMC10145165; doi:10.3389/fendo.2023.1149168)
Supplement: Supplementary file 1 [file Table_1.pdf]

SUPPLEMENTAL MATERIALS

Supplemental Table 1. Nucleotide sequences of primers used for quantitative RT-PCR detection for mRNA

Gene	Primer sequence (5' to3')	Size
ALP (mouse)	F: CCAACTCTTTTGTGCCAGAGA	21
	R: GGCTACATTGGTGTTGAGCTTTT	23
Runx2(mouse)	F: GAAATGCCTCCGCTGTTATG	20
	R: AGGTGAAACTCTTGCCTCGTC	21
Sp7(mouse)	F: ATGGCGTCCTCTCTGCTTG	19
	R: TGAAAGGTCAGCGTATGGCTT	21
Fabp4(mouse)	F: AAGGTGAAGAGCATCATAACCCT	23
	R: TCACGCCTTTCATAACACATTCC	23
Pparg(mouse)	F: ATGGTTGACACAGAGATGC	19
	R: GAATGCGAGTGGTCTTCC	18
ANGPTL2(mouse)	F: AGCCTGAGAATACCAACCGC	20
	R: CCCTTGCTTATAGGTCTCCCAG	22

Note: F, forward primer; R, reverse primer; Size, primer size.
