# Supplementary material for: Bone marrow mesenchymal stem cells derived exosomal miRNAs can modulate diabetic bone-fat imbalance
Source: Front Endocrinol (Lausanne). 2023 Apr 14;14:1149168. doi: 10.3389/fendo.2023.1149168 (PMC10145165; doi:10.3389/fendo.2023.1149168)
Supplement: Supplementary file 2 [file Image_1.pdf]

## SUPPLEMENTAL MATERIALS

**Supplemental Figure 1. Full lengeth western blot of Exosomes markers CD9, TSG101 and Calnexin**

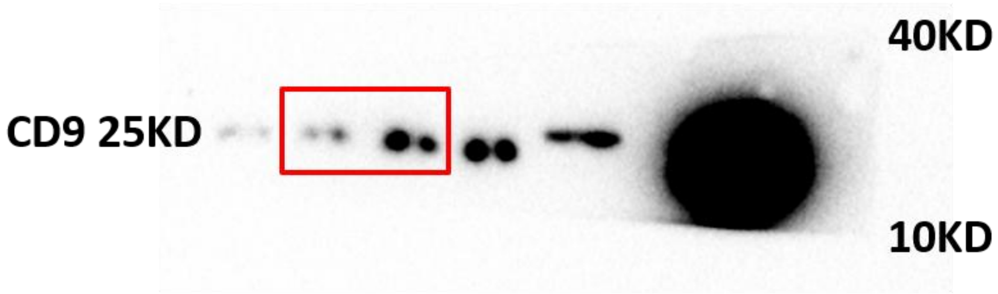


**Figure 1A. Full lengeth western blot of Exosomes markers CD9**

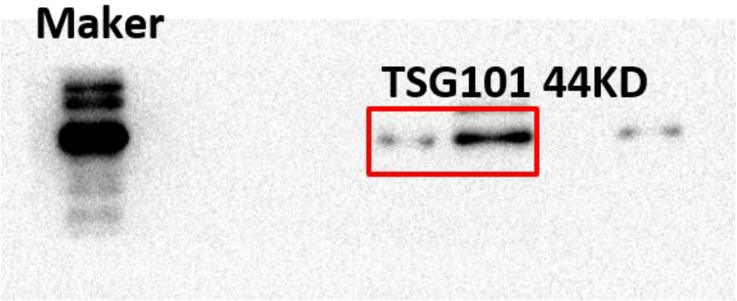


**Figure 1B. Full lengeth western blot of Exosomes markers TSG101**

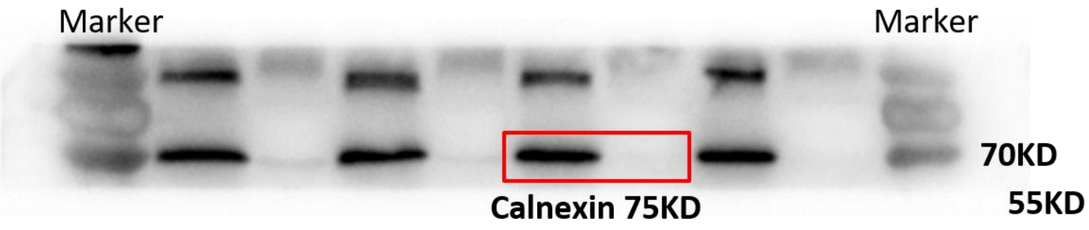


**Figure 1C. Full lengeth western blot of Exosomes markers Calnexin**
