# Supplementary material for: Bone marrow mesenchymal stem cells derived exosomal miRNAs can modulate diabetic bone-fat imbalance
Source: Front Endocrinol (Lausanne). 2023 Apr 14;14:1149168. doi: 10.3389/fendo.2023.1149168 (PMC10145165; doi:10.3389/fendo.2023.1149168)
Supplement: Supplementary file 3 [file Image_2.pdf]

SUPPLEMENTAL MATERIALS

Supplemental Figure 2. Full lengeth western blot of RUNX2, ANGPTL2, GAPDH

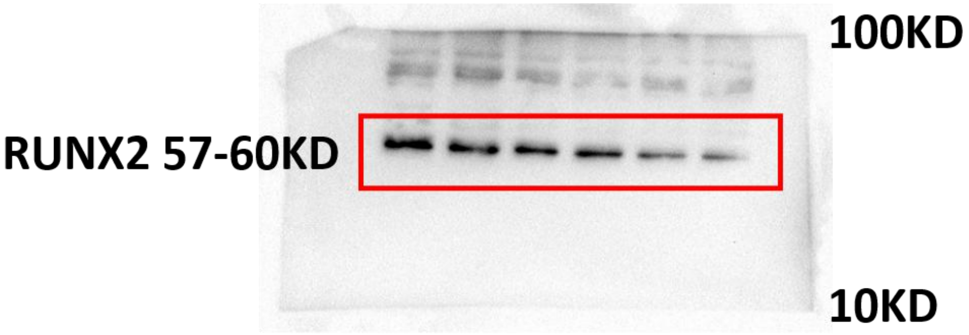

Figure 2A. Full lengeth western blot of RUNX2

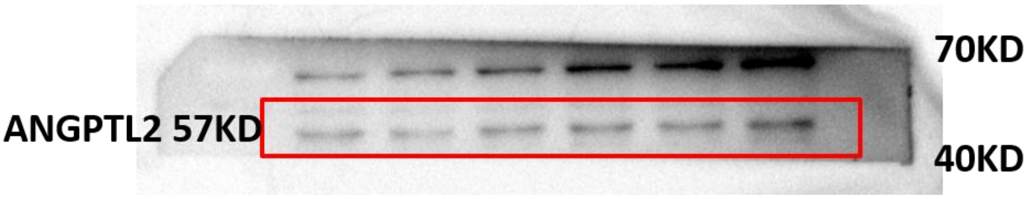

Figure 2B. Full lengeth western blot of ANGPTL2

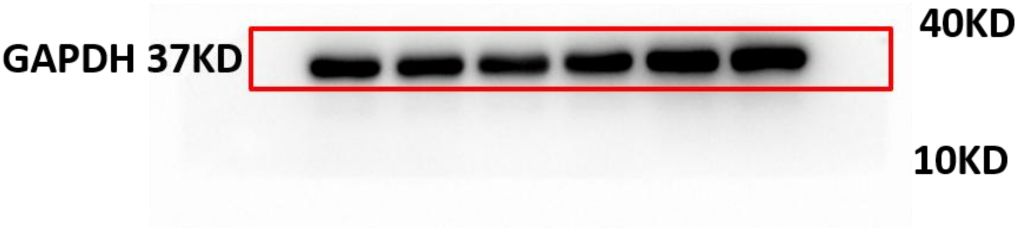

Figure 2C. Full lengeth western blot of GAPDH
