# Supplementary material for: Bone marrow mesenchymal stem cells derived exosomal miRNAs can modulate diabetic bone-fat imbalance
Source: Front Endocrinol (Lausanne). 2023 Apr 14;14:1149168. doi: 10.3389/fendo.2023.1149168 (PMC10145165; doi:10.3389/fendo.2023.1149168)
Supplement: Supplementary file 4 [file Image_3.pdf]

SUPPLEMENTAL MATERIALS

Supplemental Figure 3. Full lengeth western blot of RUNX2 and GAPDH in normal and diabetic mice

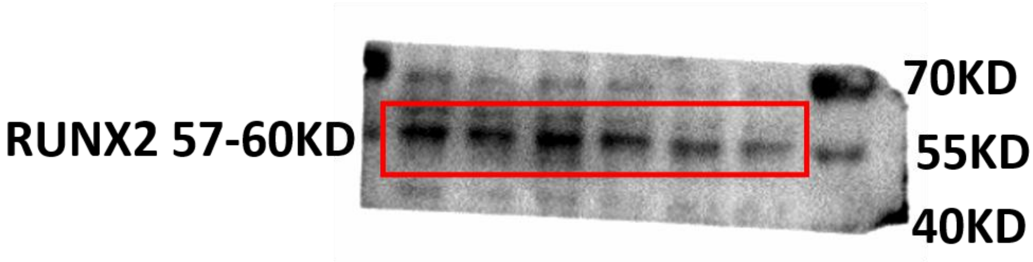

Figure 3A. Full lengeth western blot of RUNX2 in normal and diabetic mice

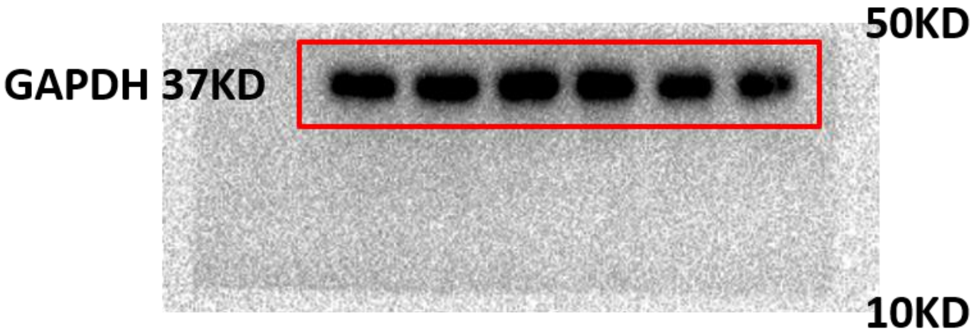

Figure 3B. Full lengeth western blot of GAPDH in normal and diabetic mice
