# Supplementary material for: Bone marrow mesenchymal stem cells derived exosomal miRNAs can modulate diabetic bone-fat imbalance
Source: Front Endocrinol (Lausanne). 2023 Apr 14;14:1149168. doi: 10.3389/fendo.2023.1149168 (PMC10145165; doi:10.3389/fendo.2023.1149168)

SUPPLEMENTAL MATERIALS

Supplemental Figure 4. Relative miR-221 expression levels in BMSCs and exosomes

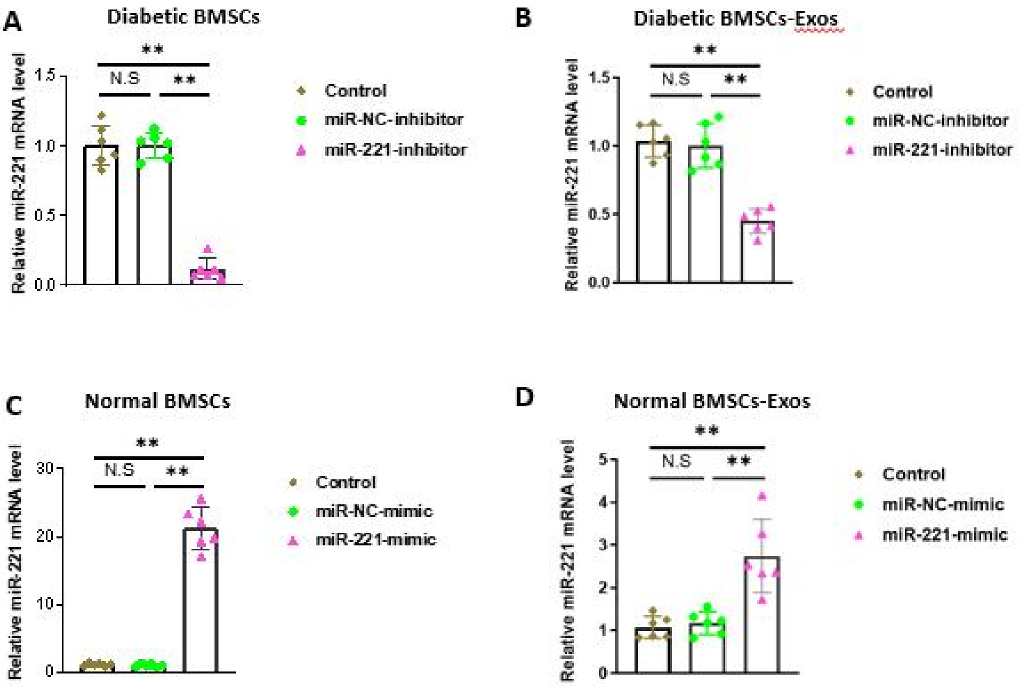

Supplement: Supplementary file 5 [file Image_4.pdf]
